# Prescription Drug Use in NMOSD: A Population-Based Study in Greece with Estimation of National Disease Administrative Prevalence

**DOI:** 10.3390/jcm14248665

**Published:** 2025-12-07

**Authors:** Christos Bakirtzis, Natalia Konstantinidou, Sotiria Stavropoulou De Lorenzo, Nikolaos Mitsoudis, Theodoros Moysiadis, Dimitrios Parisis, Marina Kleopatra Boziki, Orhan Aktas, Nikolaos Grigoriadis

**Affiliations:** 1 Second Department of Neurology, Ahepa University Hospital, Aristotle University of Thessaloniki, 54621 Thessaloniki, Greece; nataliak95@gmail.com (N.K.); iradel7714@gmail.com (S.S.D.L.); nickmitsoudis@gmail.com (N.M.); dparissis@gmail.com (D.P.); bozikim@auth.gr (M.K.B.); ngrigoriadis@auth.gr (N.G.); 2 Department of Computer Science, School of Sciences and Engineering, University of Nicosia, Nicosia 2417, Cyprus; moysiadis.t@unic.ac.cy; 3 Department of Neurology, Medical Faculty, Heinrich-Heine-Universität Düsseldorf, 40225 Düsseldorf, Germany

**Keywords:** neuromyelitis optica spectrum disorder, prevalence, immunotherapy, polypharmacy

## Abstract

**Background/Objectives**: Neuromyelitis Optica Spectrum disorder (NMOSD) is a rare, autoimmune disease of the central nervous system with a globally heterogeneous prevalence/ To estimate the prescription-based prevalence of NMOSD in Greece and determine the use of long-term immunotherapies and concomitant medications in these patients. **Methods**: We analyzed anonymized prescription records from the national prescription database, dating from 1 January 2017 to 1 May 2024. The administrative point prevalence of NMOSD in Greece on 1 January 2022 was calculated according to the national census conducted in late 2021. **Results**: We identified 219 cases of NMOSD and a 1:6.8 male-to-female ratio. The point prevalence on 1 January 2022 was 1.97 per 100,000, with the highest detected in the region of Crete, and the lowest in the regions of Eastern Macedonia and Thrace. The mean age of people with NMOSD on that date was 51.3 years old. Azathioprine was the most frequently prescribed maintenance immunotherapy, while antidepressants were the most common concomitant medications prescribed. Polypharmacy was observed in 28.9% of the identified cases. **Conclusions**: We hereby present the prescription drug use of people with NMOSD in Greece in the era just before the introduction of NMOSD-specific immunotherapy in Greece.

## 1. Introduction

Neuromyelitis Optica Spectrum Disorder (NMOSD) is an inflammatory disease of the central nervous system with a predilection for the optic nerves and the spinal cord [1]. Depending on the presence of anti-AQP4 antibodies in the serum, NMOSD is further subdivided into seropositive and seronegative forms, with approximately 80% of patients seropositive [2]. This categorization has been incorporated into the 2015 NMOSD diagnostic criteria [3]. The global epidemiological data of NMOSD are heterogeneous. A recent systematic review and meta-analysis of population-based studies around the world roughly calculated that the prevalence ranges from 0.07 to 10 per 100,000 population, with the highest prevalence rate detected in the French West Indies and South Korea and the lowest in Cuba and Australia [4]. The incidence ranged from 0.029 to 0.880 per 100,000 population, with the highest annual incidence rates observed in Sweden and the Slovak Republic and the lowest in Cuba and Australia. Previous attempts to collect global epidemiological data on NMOSD have revealed a higher prevalence of the disease in African, Asian and South American populations in comparison to Caucasian populations [5,6]. Moreover, Arnett et al. in their meta-analysis estimated that the female-to-male ratio is 9:1 [7]. A recent cross-sectional study in the U.S.A. based on electronic health record data calculated the 2022 prevalence of NMOSD to 6.88 per 100,000 population [8].

Precise prevalence estimates are essential to strengthen clinical recognition of this rare disease and to address persistent delays in diagnosis. The recent approval of multiple therapies by regulatory agencies underscores the growing importance of timely identification, as effective management is now increasingly achievable. With updated diagnostic criteria expected imminently, establishing contemporary prevalence will provide a critical reference point for guiding clinical practice and evaluating diagnostic performance. Robust epidemiological data will help clinicians achieve a comprehensive understanding of the disease and optimize patient care. In the absence of relevant epidemiological data concerning the Greek population, we aimed to estimate the prevalence of NMOSD in Greece using anonymized administrative data derived from the national prescription database. Additionally, we recorded the various long-term immunotherapy regimens used and concomitant medications, and examined polypharmacy in the identified population.

## 2. Materials and Methods

### 2.1. Data Source

In Greece, the 2015 diagnostic criteria by Wingerchuk et al. [3] are used for the diagnosis of NMOSD. Cell-based assay for anti-AQP4 antibody testing is fully reimbursed for hospitalized patients. In addition, several private laboratories perform this serology testing; therefore, people have access to anti-AQP4 antibody testing nationwide.

EOPYY, the National Organization for the Provision of Health Services, constitutes the main health care provider in Greece and is responsible for the reimbursement of medical expenses of almost all (99%) Greek citizens. Medical prescriptions are registered in a national digital database, where the International Classification of Diseases, 10th Revision (ICD-10) coding system is used. This database does not provide information such as disease onset, clinical characteristics, disease severity, or serological status.

### 2.2. Cohort Selection

Prescription records from the national prescription database, dating from 1 January 2017 to 1 May 2024, were reviewed. We only included prescriptions filled in pharmacies. Cases were classified as individuals with NMOSD when all of the following three criteria were present: (A) At least three prescriptions of either immunomodulatory or symptomatic treatment using the ICD-10 code for NMOSD (G36); (B) At least 6 months of continuous treatment with the medications mentioned above; (C) No prescriptions using the ICD-10 code for MS (G35) or sarcoidosis (D86), or prescriptions of checkpoint inhibitors either following or concomitantly with the prescriptions for NMOSD. The criteria for the algorithm were set based on the experience of previous relevant studies in MS and NMOSD using prescription datasets [8,9,10,11,12,13]. In addition, we extracted data regarding the sex, age, and residence of each identified NMOSD case.

In order to estimate the prevalence of NMOSD, data based on the population census of 2021 conducted by the Hellenic Statistical Authority (ELSTAT) were used (Hellenic Statistical Authority 2023). The administrative point prevalence on 1 January 2022 was calculated for each Greek administrative region based on the number of cases that met the criteria above. We also investigated the age distribution and sex ratio on that date. After identifying all NMOSD cases based on the three criteria above, we reviewed all their prescriptions during the study period, in order to identify both long-term immunotherapies, as well as other pharmaceutical agents used for symptomatic treatment and/or comorbid conditions. A time period of at least 6 months of continuous treatment was a prerequisite in order to record an agent. Polypharmacy was considered if at least five agents were concomitantly prescribed for a period longer than 6 months [14].

### 2.3. Ethical Approval

This retrospective cohort study was conducted after obtaining permission to use anonymized health records from the Greek Ministry of Health and in accordance with Greek legislation on personal data protection (32.1289/24 April 2019), as well as with the ethical standards established by the 1964 Declaration of Helsinki and its later amendments. Ethical approval was obtained from the local institutional ethical committee (1/12 November 2024). Informed consent of the study participants was waived by the ethical committee.

## 3. Results

During the study period, extending from 1 January 2017 until 1 May 2024, we identified 219 NMOSD cases based on prescription patterns. Of those, 191 were females, indicating a 6.8:1 female-to-male ratio. By the end of the study period, 15 NMOSD patients had died; therefore, we estimated that 204 people treated for NMOSD lived in Greece on 1 May 2024.

**Figure 1:**
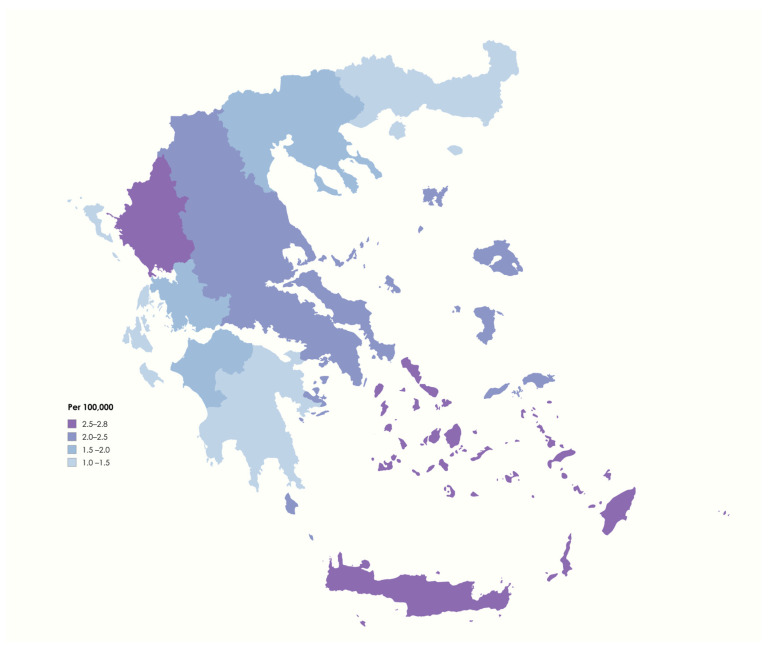
Administrative point prevalence of NMOSD in Greece on 1 January 2022 (image created with mapchart.net).

The point prevalence was calculated based on the national census conducted in late 2021, which reported a resident population in Greece on 1 January 2022 of 10,482,487 (5,356,510 females). At that point, 207 live cases treated for NMOSD were identified. Thus, our study indicates that the administrative point prevalence of NMOSD in Greece on 1 January 2022 was 1.97 per 100,000. Furthermore, a sub-analysis by region of residence was conducted. The highest point prevalence was detected in Crete (2.88 per 100,000), closely followed by Epirus (2.81 per 100,000) and Southern Aegean (2.74 per 100,000), whereas the lowest point prevalence was found in Eastern Macedonia and Thrace (1.06 per 100,000), followed by Peloponnese (1.29 per 100,000; Figure 1, Supplementary Table S1).

**Figure 2:**
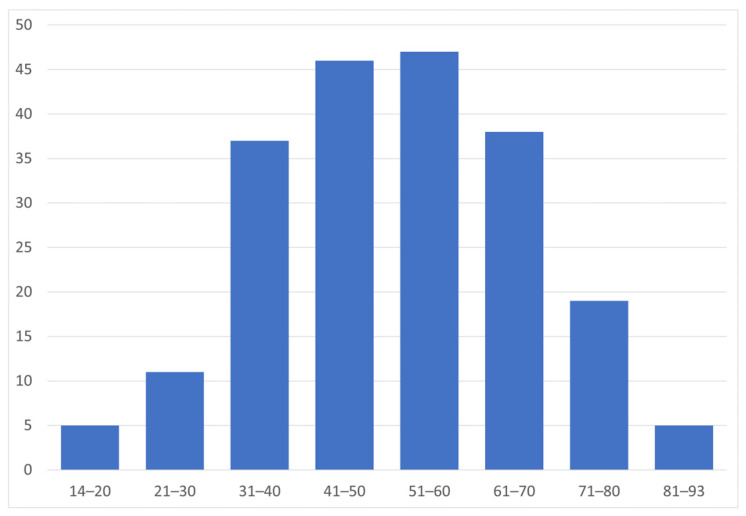
Age distribution of people treated for NMOSD (*n* = 207) on 1 January 2022.

On 1 January 2022, 87.4% of individuals treated for NMOSD were females (*n* = 181), indicating a 7.2:1 female-to-male ratio. The mean age of individuals with NMOSD on that date was 51.3 ± 15.4 years (range 14–93 years), and approximately 45% of them were aged 41–60 years (Figure 2).

In addition, we analyzed data regarding the pharmacological regimens these patients received, including both long-term immunotherapy treatment and concomitant medications. Medications prescribed for longer than six months were recorded. The type of maintenance therapy each patient received was determined according to the prescriptions reviewed on 1 January 2022. In six cases, no immunomodulatory agent was detected. It should be noted that, at that time, EMA-approved NMOSD treatments were not yet introduced in clinical practice in Greece. On 1 January 2022, among the long-term immunotherapies recorded, azathioprine was the most frequently prescribed (*n* = 97, 47.8%), followed by rituximab (*n* = 51, 24.6%). The least prescribed immunotherapeutic agents were steroids (*n* = 17, 8.2%), while mycophenolate mofetil was prescribed to 18.3% of NMOSD cases (*n* = 38). The maintenance treatment was characterized as unknown in 2.8% of our study sample.

Besides maintenance therapies, the most common pharmacological agents prescribed to the identified cases were antidepressants (34.3%), followed by antiepileptics (32.9%, of which 79.4% were gabapentinoids) and antiulcer agents (28.5%; Supplementary Table S2).

**Figure 3:**
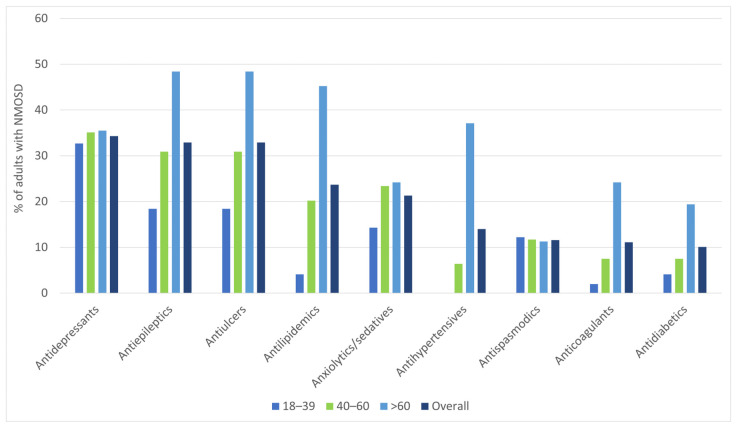
Pharmacological agents (besides immunotherapeutic drugs for maintenance therapy) that were more often prescribed (in >10% of the study sample), by age group.

In Figure 3, we present the pharmacological agents used for symptomatic treatment and for comorbid conditions that were prescribed to >10% of the study sample.

**Figure 4:**
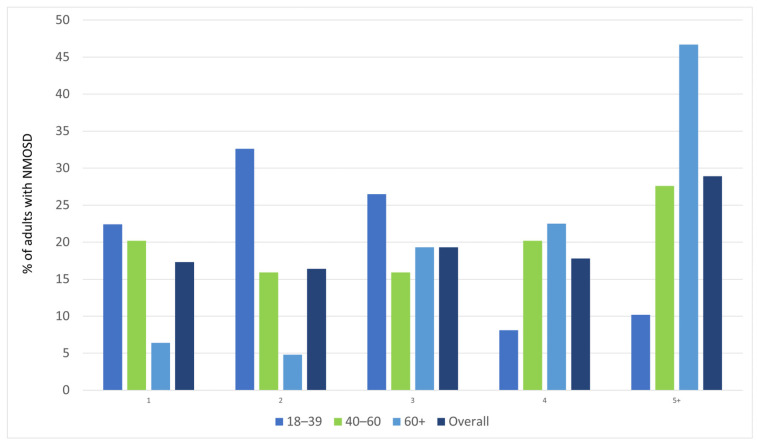
Number of pharmacological agents used concomitantly for at least 6 months, by age group.

Polypharmacy was observed in 28.9% of the identified NMOSD cases, predominantly in patients > 60 years old. Figure 4 shows the number of pharmacological agents used concomitantly for at least 6 months in various age groups of the study sample.

## 4. Discussion

In this study, we retrospectively analyzed administrative data, extracted from the national prescription database, in order to identify people treated for NMOSD in the Greek population. This study allowed an approach to the point prevalence of NMOSD in Greece, on 1 January 2022, based on the national census conducted in late 2021. According to our data based on prescription patterns, the administrative point prevalence of people treated for NMOSD was 1.97 per 100,000 population. Thus, in comparison to the global prevalence of NMOSD, ranging from 0.07 to 10 per 100,000 population, according to the most recent meta-analysis [4], Greece possesses a rather low prevalence. This finding is consistent with the fact that the prevalence of NMOSD seems to be lower in Caucasian populations [15,16]. In our sub-analysis, concerning the point prevalence of NMOSD in each Greek administrative region, southern regions closer to the African continent, like Crete and Southern Aegean, were found to have the highest point prevalence, while the lowest was recorded in northern Greece, i.e., in Eastern Macedonia and Thrace. These results seem to align with the higher NMOSD prevalence in Africans reported by various epidemiological studies, as the aforementioned southern Greek regions share similar environmental and climatic conditions with the nearby African continent [5,6,15]. People living in these Greek regions, however, are mainly of Caucasian origin, and not African; therefore, this finding is worth further examination. In addition, the results of this study highlight the need for further research examining regional variability in the availability of specialized centers, qualified medical personnel, and laboratory infrastructure, as these factors may underlie the observed heterogeneity.

The female sex seems to be an independent risk factor for NMOSD. The female-to-male ratio largely varies between the AQP4-IgG positive subgroup and its counterpart. In particular, the female-to-male ratio is 10:1 in the seropositive group, whereas in the seronegative group it is 3:1, similar to MS [17,18]. In contrast, MOG antibody-associated disease (MOGAD) has been shown to affect both sexes equally [19]. Additionally, female patients have a significantly higher likelihood of being positive for AQP4-IgG antibodies than male patients [18]. In this study, a clear female preponderance was also confirmed, although slightly lower compared to the other relevant studies. In particular, an overall 6.8:1 female-to-male ratio was estimated, whereas on 1 January 2022, the corresponding ratio was 7.2:1. AQP4-IgG status was not available in the identified cases; thus, a total ratio was calculated. Regarding the age at onset, in contrast with MS and MOGAD, which affect primarily young adults, NMOSD seems to have a higher mean age of onset, with a peak incidence around 40 years old [17]. In our study, since information related to the onset of the disease was not recorded in the national prescription database, we calculated the mean age of patients treated for NMOSD in Greece on 1 January 2022, which was found to be 51.3 years old. This finding is in accordance with the currently available global data, supporting that the onset of the disease appears mostly in middle-aged adults.

Undoubtedly, there is large heterogeneity among the epidemiological studies concerning NMOSD, due to the different inclusion criteria applied, as well as the methodology followed in each research [1]. Multi-center collaborations [17,20], hospital registries, and medical records of affiliated physicians or rehabilitation centers [15], as well as national medical databases, which are not always specific for NMOSD [8,21], were used in studies. Moreover, depending on the year and the study period, different diagnostic criteria—2006, 2015, or both—were used to identify NMOSD cases [4]. The AQP4-IgG serological status of patients with NMOSD was not available in all studies, and therefore, NMOSD cases were not always differentiated as AQP4-IgG positive or negative [22,23]. The aforementioned differences, as well as the divergence between genetic and epigenetic factors across countries and races, may be responsible for the significant variability in NMOSD prevalence worldwide [4,5,6,24,25].

NMOSD has been established as a distinct neurological disorder upon the discovery of anti-AQP4 antibodies, and a new treatment era has finally dawned after the EMA approval of targeted therapies—satralizumab, inebilizumab, eculizumab and ravulizumab [1]. During the study period, these promising NMOSD treatments were not used in clinical practice in Greece. Consequently, none of these immunomodulatory agents were identified in our research, although there may have been patients who participated in phase three clinical trials of the novel NMOSD therapies. Among the off-label classic immunotherapies recorded, azathioprine had the lion’s share, followed by rituximab. It should be noted that the use of rituximab might be underestimated in the present study, since there may have been patients who received rituximab in a number of hospitals that do not use this prescription database. Besides long-term immunotherapy, in this study, we have observed a relatively high frequency of concomitant use of other pharmacological agents—either for symptomatic treatment or for comorbid conditions. Comorbidities are often present, especially in advancing age, and seem to have a negative impact on the quality of life, the disease outcome, and may influence the choice of maintenance treatment [1,26,27]. Moreover, there may also be a possible correlation between long-term immunotherapy and the development of comorbidity [26]. In the current study, polypharmacy was observed in almost one-third of identified cases, primarily in elderly patients. Even though the use of medication for the management of disease-related symptoms or comorbid conditions may improve patients’ quality of life, it may also raise concerns such as increased complexity in everyday life, possible drug interactions and higher risk of hospitalization [14].

## 5. Limitations

The data used for this study were not retrieved from regional or single-center registries; instead, they include almost the entire Greek population; therefore, we assume that the prevalence of NMOSD was relatively accurately calculated. However, our retrospective study has a number of limitations. First, the prescription database is not an NMOSD registry; therefore, information regarding the clinical characteristics of the disease, such as the onset, severity, and clinical course, is missing in order to better characterize each case. Long-term immunotherapy is also not well defined. In some cases, an immunomodulatory agent was not detected; thus, these cases were classified as unknown treatment. These patients may have received rituximab in hospitals that do not use this prescription database, may have participated in clinical trials, or may not have received immunotherapy at all. Moreover, the AQP4-IgG serostatus, nowadays key for specific NMOSD treatment, was not available, and most probably, people with MOGAD were also included. However, the clear female preponderance in our cohort may indicate that the majority of our patients were seropositive for AQP4-IgG. Finally, it is known from MS that a relevant proportion of patients with neuroimmunological diseases do not receive specific immunotherapies. Taken together, our study reports the administrative prevalence of people treated for NMOSD and thus may reflect a (major) subset of the NMOSD population in Greece.

## 6. Conclusions

The global prevalence of NMOSD seems to be higher than it was thought to be. However, the epidemiological data regarding this disease remain limited. In the absence of such data in Greece, we hereby present the first approach to the prevalence of NMOSD in our country. Since a national NMOSD registry does not exist in Greece, we extracted our data from the national prescription database. Our results are in accordance with the general observation of both female and non-Caucasian preponderance of NMOSD, as well as the tendency of a rather late disease onset, appearing in middle-aged people. Our study will contribute to the completion of the NMOSD epidemiological world map, aiming to further define possible genetic and epigenetic risk factors, calculate health costs, organize suitable public health strategies, and finally identify those patients who will benefit from novel therapies. In order to achieve these goals, we need consistent and accurate epidemiological studies on the grounds of comparable—if not shared—methodology and data sources. Our current work highlights the imperative need for national and international NMOSD registries that provide valid, detailed clinical information and medical records to better characterize the epidemiology of the disease.

## Data Availability

Raw data used in this study are the property of the Greek Ministry of Health. Requests to access the datasets should be directed to https://www.moh.gov.gr/.

## Supplementary Materials

The following supporting information can be downloaded at: https://www.mdpi.com/article/10.3390/jcm14248665/s1, Table S1: Administrative point prevalence of NMOSD on the 1 January 2022, in each Greek region; Table S2: Pharmaceutical agents (besides maintenance immunotherapies) used by the identified NMOSD cases.
