# Supplementary material for: Prescription Drug Use in NMOSD: A Population-Based Study in Greece with Estimation of National Disease Administrative Prevalence
Source: J Clin Med. 2025 Dec 7;14(24):8665. doi: 10.3390/jcm14248665 (PMC12734201; doi:10.3390/jcm14248665)
Supplement: Supplementary file 1 [file jcm-14-08665-s001.zip › Supplementary Table S1.pdf]

Supplementary Table S1. Administrative point prevalence of NMOSD on the 1st January 2022, in each Greek region

	FEMALES		MALES		OVERALL	
**REGION**	**n**	**PREVALENCE (/100.000)**	**n**	**PREVALENCE (/100.000)**	**n**	**PREVALENCE (/100.000)**
EASTERN MACEDONIA AND THRACE	6	2.09	0	0	6	1.06
CENTRAL MACEDONIA	23	2.47	5	0.57	28	1.55
WESTERN MACEDONIA	5	3.91	1	0.78	6	2.35
EPIRUS	8	4.92	1	0.63	9	2.81
THESSALY	15	4.28	1	0.29	16	2.32
CENTRAL GREECE	10	3.94	1	0.39	11	2.16
IONIAN ISLANDS	3	2.90	0	0	3	1.46
WESTERN GREECE	10	3.09	1	0.30	11	1.69
PELOPONNESE	5	1.85	2	0.73	7	1.29

ATTICA	71	3.59	8	0.43	79	2.07
NORTH AEGEAN	2	2.05	2	2.04	4	2.05
SOUTHERN AEGEAN	8	4.90	1	0.60	9	2.74
CRETE	15	4.74	3	0.97	18	2.88
**TOTAL**	**181**	**3.37**	**26**	**0.50**	**207**	**1.97**
