# Supplementary material for: Prescription Drug Use in NMOSD: A Population-Based Study in Greece with Estimation of National Disease Administrative Prevalence
Source: J Clin Med. 2025 Dec 7;14(24):8665. doi: 10.3390/jcm14248665 (PMC12734201; doi:10.3390/jcm14248665)
Supplement: Supplementary file 1 [file jcm-14-08665-s001.zip › Supplementary Table S2.pdf]

Supplementary Table S2. Pharmaceutical agents (besides maintenance immunotherapies) used by the identified NMOSD cases.

AGE GROUP (YEARS)										
	<18 (n=2)		18-39 (n=49)		40-60 (n=94)		>60 (n=62)		Overall (n=207)	
	n	%	n	%	n	%	n	%	n	%
ANTIDEPRESSANTS	0	0	16	32.7	33	35.1	22	35.5	71	34.3
ANTI-HYPERTENSIVES	0	0	0	0	6	6.4	23	37.1	29	14
B-BLOCKERS	0	0	0	0	4	4.3	10	16.1	14	6.8
ANTISPASMODICS	0	0	6	12.2	11	11.7	7	11.3	24	11.6
ANXIOLYTICS/SEDATIVES	0	0	7	14.3	22	23.4	15	24.2	44	21.3
ANTIEPILEPTICS	0	0	9	18.4	29	30.9	30	48.4	68	32.9
ANTI-ULCERS	0	0	9	18.4	24	25	26	41.9	59	28.5
URINARY ANTISPASMODICS	0	0	3	6.1	8	8.5	7	11.3	18	8.7
BOWEL ANTISPASMODICS	0	0	0	0	0	0	1	1.6	1	0.5
PAINKILLERS/NSAIDS	0	0	1	2	7	7.5	1	1.6	9	4.3
ANTIDEMENTIA DRUGS	0	0	0	0	0	0	2	3.2	2	1
ANTITHYROIDS	0	0	3	6.1	7	7.5	7	11.3	17	8.2
ANTILIPIDEMICS	0	0	2	4.1	19	20.2	28	45.2	49	23.7

ANTIPLATELETS	0	0	0	0	3	3.2	3	4.8	6	2.9
ANTIPSYCHOTICS	0	0	4	8.2	6	6.4	6	4.8	13	6.3
BRONCHODILATORS	0	0	0	0	0	0	4	6.5	4	1.9
ANTIOSTEOPOROTICS	0	0	1	2	5	5.3	10	16.1	16	7.7
ANTICOAGULANTS	0	0	1	2	7	7.5	15	24.2	23	11.1
ANTIDIABETICS	0	0	2	4.1	7	7.5	12	19.4	21	10.1
ANTI-ALLERGIC AGENTS	0	0	1	2	1	1.1	1	1.6	3	1.5
OTHER IMMUNOMODULATORY AGENTS	0	0	4	8.2	4	4.3	4	6.5	12	5.8
ANTIPARKINSON AGENTS	0	0	3	6.1	1	1.1	2	3.2	6	2.9
DIURETICS	0	0	0	0	0	0	2	3.2	2	1
BILE ACIDS	0	0	0	0	0	0	1	1.6	1	0.5
ANTIEMETICS	0	0	0	0	0	0	1	1.6	1	0.5
ANTIANGINAL AGENTS	0	0	0	0	1	1.1	0	0	1	0.5
ANTI-GLAUCOMA AGENTS	0	0	0	0	2	2.1	1	1.6	3	1.5
ANTIESTROGENS	0	0	0	0	3	3.2	1	1.6	4	1.9
CORTICOSTEROIDS	0	0	1	2	3	3.2	1	1.6	5	2.4
PARASYMPATHOMIMETICS	0	0	0	0	2	2.1	0	0	2	1
A - BLOCKERS	0	0	1	2	2	2.1	4	6.5	7	3.4

ANTI-INFECTIVES	0	0	0	0	1	1.1	5	8.1	6	2.9
TRIPTANS	0	0	2	4.1	1	1.1	1	1.6	4	1.9
HORMONES	0	0	3	6.1	2	2.1	0	0	5	2.4
VITAMINS/MINERALS	0	0	0	0	1	1.1	1	1.6	2	1
